# Incidence and Predictors of Anticoagulant-Associated Bleeding in Patients With Venous Thromboembolism

**DOI:** 10.14740/jocmr6679

**Published:** 2026-08-26

**Authors:** Tanapun Thamgrang, Pakkapol Thiwannarak, Pornpavee Siricharoenthai, Kannadit Prayongratana, Chonlada Laoruangroj, Panachai Silpsamrit, Rattapan Lamool

**Affiliations:** a Division of Hematology, Department of Medicine, Phramongkutklao Hospital, Bangkok, Thailand; b Department of Medicine, Phramongkutklao Hospital, Bangkok, Thailand

**Keywords:** Venous thromboembolism, Anticoagulants, Bleeding, Southeast Asia, Mortality, Predictor

## Abstract

**Background:**

Data on anticoagulant-associated bleeding among patients with venous thromboembolism (VTE) in Southeast Asia are limited. We aimed to determine the incidence and predictors of anticoagulant-associated bleeding and evaluate its association with mortality.

**Methods:**

We conducted a retrospective cohort study of patients with VTE treated with anticoagulants between January 2014 and June 2024. Clinically relevant bleeding (CRB) was defined according to the International Society on Thrombosis and Haemostasis criteria. Cumulative incidence was estimated using the Aalen–Johansen method with death as a competing event, and predictors were identified using Fine–Gray sub-distribution hazard models. The association between bleeding and all-cause mortality was evaluated using time-dependent Cox proportional hazards models.

**Results:**

Among 395 patients (mean age, 60.7 ± 16.2 years; 65.3% female), 43.5% had cancer-associated thrombosis. Low-molecular-weight heparin was the most commonly prescribed initial anticoagulant (39.0%), followed by warfarin (32.7%) and direct oral anticoagulants (25.1%), of which apixaban was the most frequently used (12.7%). During a median follow-up of 0.88 years, 60 patients (15.2%) developed CRB. The 1-year cumulative incidence of CRB was 14.89% (95% confidence interval (CI), 11.09–19.23). Independent predictors of CRB included concomitant antiplatelet therapy (adjusted sub-distribution hazard ratio (aSHR), 3.48; 95% CI, 1.76–6.90), Eastern Cooperative Oncology Group performance status ≥ 2 (aSHR, 3.05; 95% CI, 1.34–6.94), body mass index (BMI) < 20 kg/m^2^ (aSHR, 2.50; 95% CI, 1.36–4.61), and age ≥ 65 years (aSHR, 1.97; 95% CI, 1.12–3.45). Both clinically relevant non-major bleeding (adjusted hazard ratio (aHR), 3.72; 95% CI, 1.55–8.92) and major bleeding (aHR, 5.62; 95% CI, 2.91–10.85) were independently associated with increased all-cause mortality.

**Conclusions:**

Anticoagulant-associated bleeding is common among Thai patients with VTE and is associated with an increased risk of all-cause mortality. Patients with advanced age, poor performance status, low BMI, or concomitant antiplatelet therapy may benefit from closer monitoring during anticoagulant treatment.

## Introduction

Venous thromboembolism (VTE), comprising deep vein thrombosis (DVT) and pulmonary embolism (PE), represents a substantial global health burden, affecting millions of patients annually [1] and ranking as the third most common cardiovascular cause of death worldwide [2]. Anticoagulant therapy is the cornerstone of VTE management; however, major bleeding (MB) remains the most frequent and serious complication of treatment. Reported incidence rates reach up to 7.22 events per 100 patient-years, depending on the class of anticoagulant used, with an associated case fatality rate of approximately 9%. Beyond its direct morbidity and mortality, MB often results in premature discontinuation of anticoagulant therapy, thereby potentially increasing the risk of recurrent thromboembolic events [3].

Ethnic differences play an important role in anticoagulant safety profiles [4–6]. Anticoagulant-associated bleeding has been reported to occur more frequently in Asian populations. Among patients with atrial fibrillation (AF), the risk of intracranial hemorrhage is higher in Asian than in non-Asian patients (1.8% vs. 0.4%) [5]. In a meta-analysis of patients with VTE comparing direct oral anticoagulants (DOACs) with vitamin K antagonists (VKAs) in Asian populations, rates of MB or clinically relevant non-major bleeding (CRNMB) differed by anticoagulant class. The absolute rate of the composite safety endpoint with DOACs was similar between Asian and non-Asian patients (7.9% vs. 7.3%), whereas the corresponding rate with VKAs was numerically higher in Asian than in non-Asian patients (11.8% vs. 9.4%) [7].

Despite these observations, there is a paucity of data on anticoagulant-associated bleeding among patients with VTE in Southeast Asia, including Thailand, as existing studies have predominantly focused on AF [8–11]. Estimating bleeding risk in VTE patients is particularly challenging because of the high prevalence of comorbidities, especially active cancer [12]. Observational studies in Asian populations consistently identify cancer as the most common risk factor for VTE [4, 6, 13] and cancer-associated thrombosis (CAT) is associated with an increased risk of bleeding and bleeding-related mortality [4]. Moreover, traditional survival analyses, such as the Kaplan–Meier method, treat death as a censoring event and may therefore overestimate the cumulative incidence of bleeding. As a result, real-world epidemiological data in Southeast Asian patients with VTE that incorporate competing-risk methodology to generate accurate bleeding risk estimates are scarce.

To address these knowledge gaps, this study aimed to determine the incidence and predictors of anticoagulant-associated bleeding in a cohort of Thai patients with VTE. By applying competing-risk analysis, we sought to provide robust real-world evidence that may offer valuable insights into risk stratification and clinical management within the Southeast Asian context.

## Materials and Methods

### Study design and setting

This retrospective cohort study included patients diagnosed with VTE who were treated at Phramongkutklao Hospital, a tertiary care center in Thailand, between January 2014 and June 2024.

### Study population

Adult patients (aged ≥ 20 years) with a first episode of objectively confirmed VTE, defined as lower-extremity DVT and/or PE, were eligible for inclusion. DVT was diagnosed using compression Doppler ultrasonography or, when clinically indicated, computed tomography venography (CTV). PE was diagnosed using computed tomography pulmonary angiography (CTPA). All included patients received anticoagulant therapy, regardless of the anticoagulant class or dosage.

Patients with VTE occurring at sites other than the lower extremities or lungs were excluded. Patients who were diagnosed with VTE at our institution but were subsequently referred to another hospital for ongoing treatment and follow-up, without any subsequent visits to our center, were also excluded. Hepatic dysfunction, active infection, and heart failure were not exclusion criteria, and patients with these conditions were eligible for inclusion.

### Objectives

The primary objective was to determine the incidence proportion, incidence rate, and 1-year cumulative incidence of clinically relevant bleeding (CRB). CRB was defined according to the International Society on Thrombosis and Haemostasis (ISTH) criteria as MB or CRNMB [14, 15].

Secondary objectives were to describe the clinical characteristics of patients who developed CRB, identify predictors of anticoagulant-associated CRB, and evaluate the association between CRB and mortality. Exploratory objectives included describing the management of CRB and estimating the incidence of recurrent VTE.

### Patient identification and data collection

Patients were identified through the hospital electronic medical record (EMR) system by searching for International Classification of Diseases, 10th Revision (ICD-10) codes corresponding to DVT (I80, I82) and PE (I26) during the study period. Each identified case was individually reviewed to confirm the diagnosis of VTE, and only patients who met the predefined inclusion and exclusion criteria were included in the final analysis.

Baseline characteristics—including age, sex, comorbidities, type of VTE, provoking factors, and laboratory parameters—were collected using the Research Electronic Data Capture (REDCap) electronic data capture system. Dates of anticoagulant initiation and discontinuation, as well as the date of last follow-up, were recorded for time-to-event analyses. Bleeding events and recurrent thrombotic events occurring during follow-up were systematically documented.

A body mass index (BMI) cutoff of < 20 kg/m^2^ was selected based on previous studies suggesting an increased risk of MB among patients receiving anticoagulant therapy with a BMI < 20 kg/m^2^ [16, 17].

Active cancer was defined as cancer diagnosed or treated within the previous 6 months; recurrent, regionally advanced, or metastatic disease; or hematological malignancy not in complete remission [18]. Anemia was defined as a hemoglobin level < 13 g/dL in males and < 12 g/dL in females. Chronic kidney disease (CKD) was defined by the presence of kidney damage, an estimated glomerular filtration rate (eGFR) < 60 mL/min/1.73 m^2^, or a documented physician diagnosis of CKD in the medical record.

Only the first bleeding event per patient during the study period was included in the analysis. Recurrent thrombotic events were defined as objectively confirmed recurrent VTE based on standard imaging modalities (e.g., compression ultrasonography or computed tomography) during follow-up.

### Anticoagulant classification and follow-up strategy

The anticoagulant reported in the baseline characteristics represents the initial intended treatment strategy prescribed during the primary treatment phase of VTE [19]. Dosages reflect those used during this initial therapeutic period. Patients receiving warfarin, edoxaban, or dabigatran with bridging or lead-in heparin therapy were classified according to the specific oral anticoagulant received. Those treated exclusively with heparin were categorized as receiving heparin monotherapy. Patients who initially received heparin and were subsequently transitioned to a DOAC (including apixaban or rivaroxaban) as part of their planned primary treatment were classified in the DOAC group.

Follow-up commenced at initiation of anticoagulant therapy and continued until the first occurrence of a bleeding event, death, or loss to follow-up, whichever occurred first. To preserve the validity of risk estimates associated with the initial treatment strategy, patients were censored at the time of any deviation from the primary regimen.

Deviations were defined as permanent discontinuation of anticoagulation; transition from the primary treatment phase to extended-phase (secondary prevention) therapy involving a change in anticoagulant class or a planned dose reduction; or any switch between different anticoagulant classes (such as routine clinical transitions between VKAs and DOACs, or transitions driven by non-clinical reasons like medication cost, patient preference, or drug availability). Additionally, any switch between different anticoagulant classes driven by a non-bleeding clinical event (e.g., recurrent thrombosis) resulted in censoring at the time of the switch. Conversely, switching between agents within the same anticoagulant class for clinical indications (e.g., intolerance or adverse effects) was not considered a deviation. In such cases, patients remained classified within the same anticoagulant class, and dosage information reflected the regimen administered until a subsequent change in treatment occurred.

For secondary survival analysis, overall survival (OS) was defined as the time from the initiation of anticoagulant therapy to death from any cause or last follow-up, whichever occurred first.

### Statistical analysis

Categorical variables are presented as frequencies and percentages and were compared using Fisher’s exact test. Continuous variables are presented as mean ± standard deviation (SD) or median (interquartile range (IQR)), as appropriate, and were compared using the independent *t*-test or the Wilcoxon rank-sum test.

Cumulative incidence functions were estimated using the Aalen–Johansen method within a competing-risk framework. For overall CRB, MB and CRNMB were treated as failure events, with death considered a competing event. For analyses of MB alone, CRNMB and death were treated as competing events.

Missing data among variables planned for inclusion in the multivariable model were assessed using Little’s test for missing completely at random (MCAR). As the test was statistically significant, data were assumed to be missing at random. Missing baseline variables (BMI, Eastern Cooperative Oncology Group (ECOG) performance status, and hemoglobin level) were handled using multiple imputation by chained equations (MICE) with 50 imputations. All variables included in the multivariable model, as well as the Nelson–Aalen cumulative hazard estimator and bleeding events, were incorporated into the imputation model. The largest fraction of missing information (FMI) was 0.38, with a relative increase in variance (RVI) of 0.19. With 50 imputations, the relative efficiency exceeded 99%, indicating adequate precision. Pooled estimates were derived using Rubin’s rules.

Predictors of CRB were evaluated using Fine and Gray competing-risk regression models. Variables with P values < 0.20 in univariable analysis were considered candidates for inclusion in the multivariable model. The significance of predictors in the pooled multivariable models was assessed using Wald tests according to Rubin’s rules. The proportional sub-distribution hazards assumption was evaluated for all covariates, and no significant violations were detected.

The association between bleeding events and all-cause mortality was evaluated using time-dependent Cox proportional hazards regression models. Bleeding events were modeled as time-dependent covariates. Candidate variables with a P value < 0.20 in the univariable time-dependent Cox regression were included in the initial multivariable model. Model simplification was performed via backward elimination guided by the joint Wald test. Non-significant covariates were sequentially removed if their joint deletion did not significantly alter model fit (P value ≥ 0.05). Other clinically important risk factors were retained in the final multivariable model regardless of statistical significance. The proportional hazards assumption was assessed using Schoenfeld residuals.

Sensitivity analyses were performed after excluding patients who received low-molecular-weight heparin (LMWH) at intermediate or prophylactic doses, as well as those receiving reduced-dose DOACs, defined as dabigatran 110 mg twice daily, apixaban 2.5 mg twice daily, rivaroxaban 10 mg once daily, or edoxaban 30 mg once daily. The cumulative incidence of CRB was re-estimated in the restricted cohort. The Fine–Gray regression analysis was then repeated using the same predictors included in the final multivariable model to assess the robustness of the identified predictors of CRB.

All analyses were performed using Stata version 17.0 (StataCorp LLC, College Station, TX, USA). Two-sided P values < 0.05 were considered statistically significant.

### Sample size calculation

The sample size was estimated based on the GARFIELD-VTE study [6], which reported a bleeding incidence of 9.6 per 100 patient-years in Asian populations. Assuming a median follow-up of 1 year, this corresponded to an anticipated cumulative incidence of 9.6%. Using a 95% confidence level and a margin of error of 3%, a minimum of 371 patients was required. After accounting for an anticipated 5% loss to follow-up, the final target sample size was set at 391 patients. This sample size was expected to yield an adequate number of events to support multivariable analyses of 3–4 independent predictors, maintaining approximately 10 events per variable [20].

### Ethical considerations

This study was approved by the Institutional Review Board of the Royal Thai Army Medical Department (Approval Number IRBRTA 0937/2567). The study was conducted in accordance with the principles of the Declaration of Helsinki and adhered to the International Conference on Harmonization Good Clinical Practice (ICH-GCP) guidelines.

## Results

A total of 910 patients with VTE were identified through ICD-10 screening. Of these, 515 were excluded for the following reasons: pediatric age (n = 14), refusal of anticoagulation (n = 32), receipt of thromboprophylaxis rather than therapeutic anticoagulation (n = 44), venous insufficiency without confirmed VTE (n = 38), treatment at another hospital (n = 110), and unusual-site VTE (n = 277).

The final cohort comprised 395 patients included in the analysis. During follow-up, 60 patients developed CRB, representing an incidence proportion of 15.2% (95% confidence interval (CI), 11.8–19.1). Of these, 32 were MB events (8.1%) and 28 were CRNMB events (7.1%) (Supplementary Material 1, jocmr.elmerjournals.com).

### Baseline characteristics

The mean age of the cohort was 60.7 ± 16.2 years and was significantly higher among patients who developed CRB than among those who did not (67.4 ± 15.7 vs. 59.5 ± 16.0 years; P = 0.001). A greater proportion of patients aged ≥ 65 years was observed in the bleeding group (37 (61.7%) vs. 122 (36.4%); P < 0.001). Most patients were female (258 (65.3%)), with a similar distribution between groups. The median BMI was 23.44 kg/m^2^ (IQR, 20.70–26.16), based on available data for 332 patients. A BMI < 20 kg/m^2^ was more frequent in the bleeding group compared with the non-bleeding group (15 (31.9%) vs. 49 (17.2%); P = 0.027). An ECOG performance status ≥ 2 was also more common among patients with CRB (7 (18.0%) vs. 8 (3.5%); P = 0.002).

Hypertension was the most prevalent comorbidity (140 (35.4%)). Cerebrovascular disease was more frequent in the bleeding group. Active cancer was present in 172 patients (43.5%), the majority of whom had solid malignancies (159 (92.4%)).

Initial concomitant antiplatelet therapy included aspirin (21 (5.3%)), clopidogrel (3 (0.8%)), and dual antiplatelet therapy (7 (1.8%)). The proportion of patients receiving antiplatelet therapy was significantly higher in the bleeding group than in the non-bleeding group (P = 0.002). Nonsteroidal anti-inflammatory drug (NSAID) use was reported in two patients (0.5%). Use of oral contraceptives or hormone replacement therapy was recorded in eight patients (2.0%).

The distribution of VTE presentation was as follows: PE alone in 191 patients (48.4%), DVT alone in 146 (37.0%), and concomitant DVT and PE in 58 (14.7%). Regarding VTE classification, 172 patients (43.5%) had CAT, 80 (20.3%) had provoked VTE (including transient and persistent risk factors apart from active cancer), and 143 (36.2%) had unprovoked VTE. The distribution of VTE presentation and classification did not differ significantly between the bleeding and non-bleeding groups.

LMWH was the most frequently prescribed initial anticoagulant, used in 154 patients (39.0%), followed by warfarin in 129 (32.7%). DOACs were prescribed in 99 patients (25.1%), with apixaban being the most commonly used DOAC (50 (12.7%)).

Additional baseline characteristics and laboratory parameters at the time of VTE diagnosis are summarized in Table 1. Detailed information regarding anticoagulant dosing in the study population is provided in Table 2. The distribution of primary cancer sites is presented in Table 3.

**Table 1:** Baseline Characteristics

Characteristics	Total	Clinically relevant bleeding	No bleeding	P value
N (%)	395 (100)	60 (15.19)	335 (84.81)	
Age, years	60.71 ± 16.22	67.39 ± 15.67	59.52 ± 16.04	0.001
≥ 65, n (%)	159 (40.25)	37 (61.67)	122 (36.42)	< 0.001
Sex, n (%)				1.000
Female	258 (65.32)	39 (65.00)	219 (65.37)	
Male	137 (34.68)	21 (35.00)	116 (34.63)	
Weight, median (IQR), kg	59.5 (50.1–70.0), n = 353	53 (48–66), n = 51	60 (52–70), n = 302	0.021
Height, median (IQR), cm	160 (155–167), n = 347	160 (155–165.5), n = 52	160 (155–167), n = 295	0.970
BMI, median (IQR), kg/m^2^	23.44 (20.70–26.16), n = 332	22.21 (19.72–24.80), n = 47	23.61 (20.82–26.35), n = 285	0.021
< 18.5	38 (11.45)	7 (14.89)	31 (10.88)	0.457
< 20	64 (19.28)	15 (31.91)	49 (17.19)	0.027
ECOG^a^ performance status, n (%)				0.002
0–1	255 (94.44)	32 (82.05)	223 (96.54)	
≥ 2	15 (5.56)	7 (17.95)	8 (3.46)	
Comorbidity, n (%)				
Diabetes mellitus	83 (21.01)	14 (23.33)	69 (20.60)	0.609
Hypertension	140 (35.44)	27 (45.00)	113 (33.73)	0.107
Coronary artery disease	11 (2.78)	4 (6.67)	7 (2.09)	0.069
Cerebrovascular disease	18 (4.56)	7 (11.67)	11 (3.28)	0.011
Chronic liver disease/cirrhosis	9 (2.28)	1 (1.67)	8 (2.39)	1.000
Chronic kidney disease	31 (7.85)	6 (10.00)	25 (7.46)	0.445
Peripheral arterial disease	4 (1.01)	1 (1.67)	3 (0.90)	0.484
Active cancer, n (%)	172 (43.54)	26 (43.33)	146 (43.58)	1.000
Solid malignancy	159 (92.44)	24 (92.31)	135 (92.47)	
Hematologic malignancy	13 (7.56)	2 (7.69)	11 (7.53)	
Alcohol use, n (%)	5 (1.37)	2 (3.33)	3 (0.90)	0.167
Medication, n (%)				
Antiplatelet				0.002
Aspirin (ASA)	21 (5.32)	8 (13.33)	13 (3.88)	
Clopidogrel	3 (0.76)	1 (1.67)	2 (0.60)	
Dual antiplatelets	7 (1.77)	3 (5.00)	4 (1.19)	
NSAIDs	2 (0.51)	1 (1.67)	1 (0.30)	0.281
Oral contraceptives or hormone therapy, n (%)	8 (2.03)	0 (0.00)	8 (2.39)	0.613
Estrogen	1 (0.25)	0 (0.00)	1 (0.30)	
Progestin	0 (0.00)	0 (0.00)	0 (0.00)	
Estrogen and progestin	5 (1.26)	0 (0.00)	5 (1.49)	
Androgen	0 (0.00)	0 (0.00)	0 (0.00)	
Unknown	2 (0.51)	0 (0.00)	2 (0.60)	
History of surgery within 3 months before initiation of anticoagulation, n (%)	59 (14.94)	8 (13.33)	51 (15.22)	0.845
Bleeding risk of procedure^b^, n (%)				0.407
Low to moderate bleeding risk	44 (74.58)	5 (62.50)	39 (76.47)	
High bleeding risk	15 (25.42)	3 (37.50)	12 (23.53)	
History of bleeding within 3 months before initiation of anticoagulation, n (%)				0.082
Clinically relevant non-major bleeding	8 (2.03)	1 (1.67)	7 (2.09)	
Major bleeding	6 (1.52)	3 (5.00)	3 (0.90)	
Type of VTE, n (%)				0.106
DVT	146 (36.96)	22 (36.67)	124 (37.01)	
PE	191 (48.35)	24 (40.00)	167 (49.85)	
Both DVT and PE	58 (14.68)	14 (23.33)	44 (13.13)	
VTE classification, n (%)				0.966
Unprovoked	143 (36.20)	21 (35.00)	122 (36.42)	
Provoked^c^	80 (20.25)	13 (21.67)	67 (20.00)	
Cancer-associated thrombosis (CAT)	172 (43.54)	26 (43.33)	146 (43.58)	
Thrombophilia workup, n (%)				1.000
Normal/negative	145 (36.71)	22 (36.67)	123 (36.72)	
Protein C deficiency	1 (0.25)	0 (0.00)	1 (0.30)	
Antithrombin deficiency	1 (0.25)	0 (0.00)	1 (0.30)	
Antiphospholipid syndrome (APS)	14 (3.54)	2 (3.33)	12 (3.58)	
No workup	234 (59.24)	36 (60.00)	198 (59.10)	
Anticoagulants, n (%)				
Individual agents				0.071
Warfarin	129 (32.66)	19 (31.67)	110 (32.84)	
UFH	13 (3.29)	3 (5.00)	10 (2.99)	
LMWH	154 (38.99)	25 (41.67)	129 (38.51)	
Apixaban	50 (12.66)	3 (5.00)	47 (14.03)	
Rivaroxaban	28 (7.09)	3 (5.00)	25 (7.46)	
Edoxaban	17 (4.30)	5 (8.33)	12 (3.58)	
Dabigatran	4 (1.01)	2 (3.33)	2 (0.60)	
Type of anticoagulant				0.628
DOACs	99 (25.06)	13 (21.67)	86 (25.67)	
Non-DOACs	296 (74.94)	47 (78.33)	249 (74.33)	
Laboratory at VTE diagnosis				
Hb^d^, g/dL	11.05 ± 2.18	10.57 ± 2.14	11.14 ± 2.18	0.067
Anemia, n (%)	253 (69.13)	45 (77.59)	208 (67.53)	0.163
< 10, n (%)	117 (31.97)	24 (41.38)	93 (30.19)	0.124
Platelets^d^, median (IQR), /µL	250,500 (187,000–334,000)	240,500 (175,000–369,000)	252,500 (189,500–328,000)	0.921
Creatinine^e^, median (IQR), mg/dL	0.78 (0.61–1.04)	0.79 (0.6–1.1)	0.77 (0.61–1)	0.396
eGFR^e^, median (IQR), mL/min/1.73 m^2^	92.28 (66.85–105.42)	80.77 (55.10–98.20)	93.56 (71.18–106.08)	0.010
≥ 30, n (%)	341 (94.72)	53 (91.38)	288 (95.36)	
≥ 15 to < 30, n (%)	16 (4.44)	4 (6.90)	12 (3.97)	
< 15, n (%)	3 (0.83)	1 (1.72)	2 (0.66)	

^a^ECOG performance status was available for 270 patients. ^b^Procedural bleeding risk was stratified according to ISTH guidance statements. ^c^Provoking factors other than cancer-associated thrombosis. ^d^Hemoglobin levels and platelet counts were available for 366 patients. ^e^Serum creatinine was available for 360 patients. BMI: body mass index; NSAID: nonsteroidal anti-inflammatory drug; VTE: venous thromboembolism; DVT: deep vein thrombosis; PE: pulmonary embolism; Hb: hemoglobin; eGFR: estimated glomerular filtration rate; LMWH: low-molecular-weight heparin; UFH: unfractionated heparin; DOAC: direct oral anticoagulant; IQR: interquartile range; ECOG: Eastern Cooperative Oncology Group.

**Table 2:** Doses of Anticoagulants Used in the Study Population

Anticoagulants	Total	Clinically relevant bleeding	No bleeding	P value
N (%)	395 (100)	60 (15.19)	335 (84.81)	
Warfarin, n (%)	129 (32.66)	19 (31.67)	110 (32.84)	
TTR^a^, median (IQR), %	30.0 (16.8–50.0), n = 100	36.8 (5.0–60.0), n = 15	28.0 (19.0–50.0), n = 85	0.831
UFH, n (%)	13 (3.29)	3 (5.00)	10 (2.99)	
LMWH, n (%)	154 (38.99)	25 (41.67)	129 (38.51)	
Therapeutic dose	150 (37.97)	25 (41.67)	125 (37.31)	
Intermediate dose	2 (0.51)	0 (0.00)	2 (0.6)	
Prophylaxis dose (e.g., enoxaparin 40 mg daily)	2 (0.51)	0 (0.00)	2 (0.6)	
DOACs, n (%)	99 (25.06)	13 (21.67)	86 (25.67)	
Dabigatran 110 mg twice daily	2 (0.51)	1 (1.67)	1 (0.30)	
Dabigatran 150 mg twice daily	2 (0.51)	1 (1.67)	1 (0.30)	
Apixaban 2.5 mg twice daily	15 (3.80)	0 (0.00)	15 (4.48)	
Apixaban 5 mg twice daily	34 (8.61)	3 (5.00)	31 (9.25)	
Apixaban 10 mg twice daily	1 (0.25)	0 (0.00)	1 (0.30)	
Rivaroxaban 20 mg once daily	19 (4.81)	1 (1.67)	18 (5.37)	
Rivaroxaban 15 mg twice daily	3 (0.76)	1 (1.67)	2 (0.60)	
Rivaroxaban 10 mg once daily	6 (1.52)	1 (1.67)	5 (1.49)	
Edoxaban 60 mg once daily	12 (3.04)	3 (5.00)	9 (2.69)	
Edoxaban 30 mg once daily	5 (1.27)	2 (3.33)	3 (0.90)	

^a^TTR (%) was calculated using the traditional (direct) method based on the proportion of INR values within the therapeutic range for up to 1 year after warfarin initiation. LMWH: low-molecular-weight heparin; UFH: unfractionated heparin; DOAC: direct oral anticoagulant; TTR: time in therapeutic range.

**Table 3:** Baseline Characteristics of Patients With Active Cancer According to Clinically Relevant Bleeding Status

Primary cancer site	Total (N = 172)	Clinically relevant bleeding (N = 26)	No bleeding (N = 146)
Gynecological	47 (27.33)	11 (42.31)	36 (24.66)
Lung	34 (19.77)	4 (15.38)	30 (20.55)
Urological^a^	17 (9.88)	2 (7.69)	15 (10.27)
Colorectal	14 (8.14)	1 (3.85)	13 (8.90)
Breast	11 (6.40)	2 (7.69)	9 (6.16)
Lymphoma^b^	11 (6.40)	1 (3.85)	10 (6.85)
Cholangiocarcinoma	6 (3.49)	1 (3.85)	5 (3.42)
Pancreatic	6 (3.49)	0 (0.00)	6 (4.11)
Central nervous system^c^	4 (2.33)	1 (3.85)	3 (2.05)
Gastric	3 (1.74)	0 (0.00)	3 (2.05)
Head and neck	3 (1.74)	2 (7.69)	1 (0.68)
Thyroid	3 (1.74)	0 (0.00)	3 (2.05)
Hepatocellular	3 (1.74)	0 (0.00)	3 (2.05)
Multiple myeloma	2 (1.16)	1 (3.85)	1 (0.68)
Bone and soft tissue sarcoma	2 (1.16)	0 (0.00)	2 (1.37)
Esophageal	1 (0.58)	0 (0.00)	1 (0.68)
Germ cell	1 (0.58)	0 (0.00)	1 (0.68)
Gallbladder	1 (0.58)	0 (0.00)	1 (0.68)
Two primary cancers^d^	1 (0.58)	0 (0.00)	1 (0.68)
Primary peritoneal cancer	1 (0.58)	0 (0.00)	1 (0.68)
Unknown primary	1 (0.58)	0 (0.00)	1 (0.68)

^a^Bladder, renal cell carcinoma, and prostate cancer. ^b^Diffuse large B-cell lymphoma, follicular lymphoma, extranodal marginal zone lymphoma, primary mediastinal B-cell lymphoma, and small lymphocytic lymphoma. ^c^Primary brain tumors and central nervous system germinoma. ^d^Synchronous cecal and ovarian cancers.

### Characteristics of CRB

Over a median follow-up of 0.88 years (95% CI, 0.69–1.21), 60 patients experienced CRB, including 32 patients (53.3%) with MB and 28 patients (46.7%) with CRNMB.

Across 499.38 patient-years of follow-up, the incidence rate of CRB was 12.02 per 100 patient-years (95% CI, 9.33–15.47). The incidence rate of first MB was 6.41 per 100 patient-years (95% CI, 4.53–9.06), while that of first CRNMB was 5.61 per 100 patient-years (95% CI, 3.87–8.12).

Most bleeding events occurred within the first year after initiation of anticoagulation. The cumulative incidence of CRB at 1 and 2 years was 14.89% (95% CI, 11.09–19.23) and 19.36% (95% CI, 14.59–24.64), respectively.

**Figure 1:**
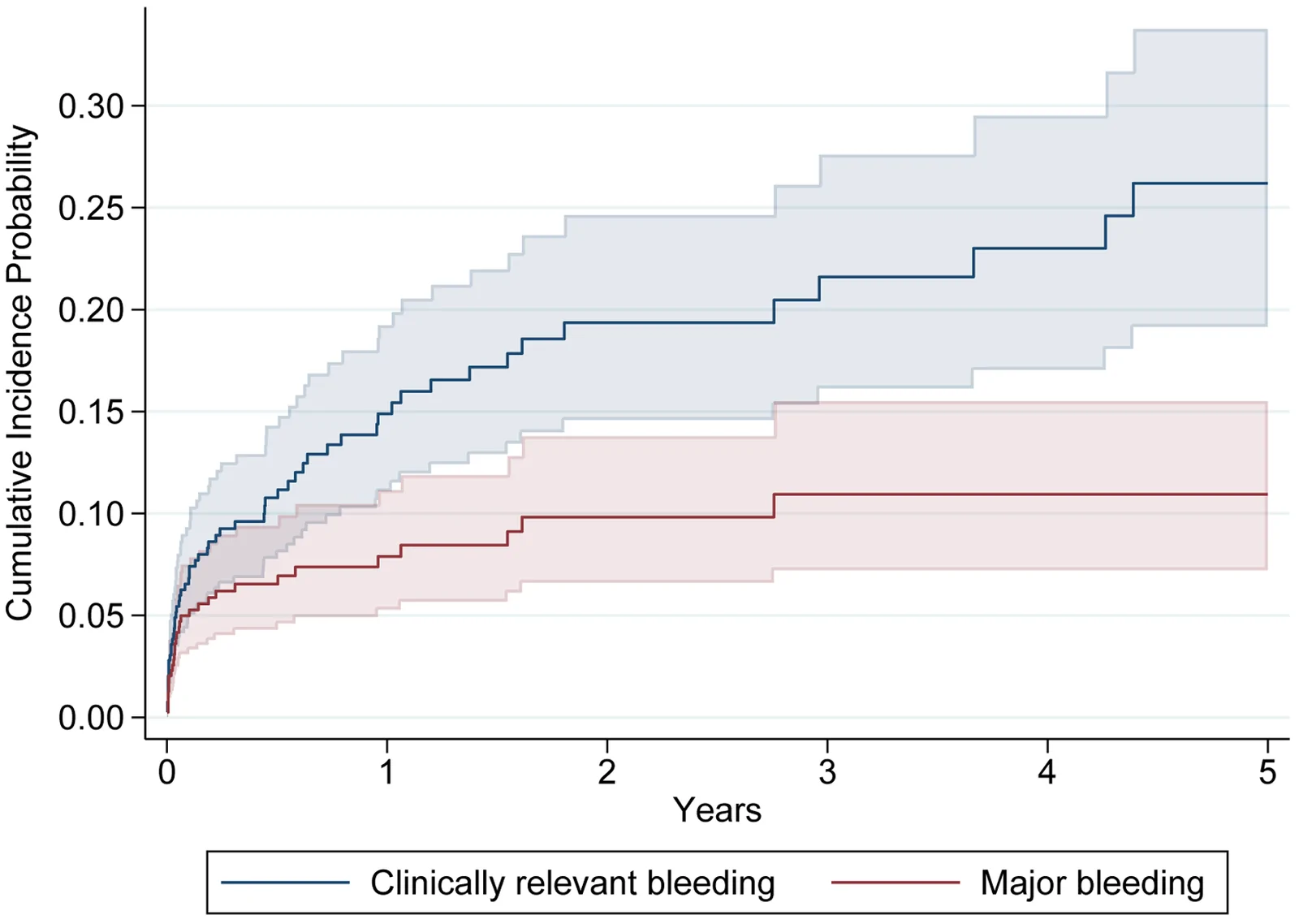
Cumulative incidence of clinically relevant bleeding and major bleeding estimated using the Aalen–Johansen method. For CRB, death was treated as a competing event. For MB, CRNMB and death were treated as competing events; therefore, the cumulative incidence represents the probability of experiencing MB as the first bleeding event. Shaded areas represent the 95% confidence intervals. CRB: clinically relevant bleeding; MB: major bleeding; CRNMB: clinically relevant non-major bleeding.

For MB, the cumulative incidence of first MB was 7.89% (95% CI, 5.29–11.15) at 1 year and 9.83% (95% CI, 6.61–13.79) at 2 years (Fig. 1).

The median time to first bleeding event was 1.95 months (IQR, 0.33–10.46). Patients who developed MB experienced bleeding earlier than those with CRNMB (median, 0.59 months (IQR, 0.16–4.88) vs. 5.98 months (IQR, 1.20–13.32); P = 0.018).

Most bleeding events occurred in the outpatient setting (40 patients (66.7%)); however, 24 patients (60.0% of outpatient events) subsequently required hospitalization. Gastrointestinal (GI) bleeding was the most common site, including upper GI bleeding in 16 patients (26.7%) and lower GI bleeding in nine (15.0%). Skin and musculoskeletal bleeding occurred in 16 patients (26.7%). Central nervous system (CNS) bleeding was observed in six patients (10.0%) and accounted for 18.8% of all MB events.

A total of 28 patients (46.7%) required transfusion of ≥ 2 units of packed red blood cells. Surgical or procedural hemostasis was required in 24 patients (40.0%), more frequently among those with MB than among those with CRNMB (19 (59.4%) vs. 5 (17.9%); P = 0.001). More than half of patients with bleeding (31 (51.7%)) permanently discontinued anticoagulation. Permanent discontinuation occurred in 20 patients (62.5%) with MB and 11 (39.3%) with CRNMB, although the difference was not statistically significant (P = 0.120). Among patients with MB, 14 (43.8%) received a specific reversal agent.

Detailed characteristics of bleeding events and their management are summarized in Table 4 and Supplementary Materials 2 and 3 (jocmr.elmerjournals.com).

**Table 4:** Clinical Characteristics of Patients With Clinically Relevant Bleeding

Characteristics	Total bleeding (N = 60)	Major bleeding (N = 32)	Clinically relevant non-major bleeding (N = 28)	P value
Time to first bleeding event, median (IQR), months	1.95 (0.33–10.46)	0.59 (0.16–4.88)	5.98 (1.20–13.32)	0.018
INR at bleeding event in warfarin users, median (IQR)	2.66 (1.97–5.31), n = 17	3.45 (2.11–5.31), n = 9	2.33 (1.57–4.30), n = 8	0.370
Site of bleeding^a^, n (%)				
Upper gastrointestinal	16 (26.67)	10 (31.25)	6 (21.43)	0.559
Lower gastrointestinal	9 (15.00)	5 (15.63)	4 (14.29)	1.000
Central nervous system	6 (10.00)	6 (18.75)	0 (0.00)	0.026
Musculoskeletal/skin	16 (26.67)	7 (21.88)	9 (32.14)	0.397
Respiratory	2 (3.33)	1 (3.13)	1 (3.57)	1.000
Genitourinary	7 (11.67)	3 (9.38)	4 (14.29)	0.695
Other	5 (8.33)	1 (3.13)	4 (14.29)	0.175
Outpatient bleeding, n (%)	40 (66.67)	19 (59.38)	21 (75.00)	0.274
Bleeding requiring hospital admission, n (%)	24 (60.00)	18 (94.74)	6 (28.57)	< 0.001
All-cause mortality, n (%)	18 (30.00)	12 (37.50)	6 (21.43)	0.259
Bleeding-related mortality^b^, n (%)	1 (5.56)	1 (8.33)	0 (0.00)	

^a^Patients may have experienced bleeding at multiple sites. Percentages are calculated based on the total number of patients in each group, and the sum of patients by site may exceed the total N in each category. ^b^Percentages were calculated using the number of patients in each column as the denominator. INR: international normalized ratio; IQR: interquartile range.

### Predictors of CRB

The results of the univariable and multivariable competing-risk regression analyses for CRB are presented in Table 5.

**Table 5:** Univariable and Multivariable Competing Risk Regression Analysis of Clinically Relevant Bleeding

Predictor	Univariable model		Multivariable model^c^	
	Crude SHR (95% CI)	P value	Adjusted SHR (95% CI)	P value
Age ≥ 65 years	2.51 (1.50–4.20)	< 0.001	1.97 (1.12–3.45)	0.018
Male	1.03 (0.60–1.74)	0.926		
BMI^a^ < 20 kg/m^2^	2.20 (1.25–3.88)	0.007	2.50 (1.36–4.61)	0.003
ECOG^a^ ≥ 2	3.86 (1.80–8.28)	0.001	3.05 (1.34–6.94)	0.008
Diabetes mellitus	1.11 (0.61–2.00)	0.738		
Hypertension	1.50 (0.90–2.49)	0.116		
Coronary artery disease	2.92 (0.99–8.63)	0.052		
Cerebrovascular disease	2.92 (1.44–5.92)	0.003		
Chronic liver disease/cirrhosis	0.76 (0.11–5.07)	0.778		
Chronic kidney disease	1.30 (0.59–2.88)	0.513		
Peripheral arterial disease	1.25 (0.19–8.26)	0.817		
Active cancer	1.15 (0.69–1.92)	0.589		
Antiplatelet use	3.54 (1.95–6.43)	< 0.001	3.48 (1.76–6.90)	< 0.001
History of previous bleeding^b^	1.58 (0.61–4.08)	0.347		
Non-DOAC therapy	1.54 (0.84–2.82)	0.163	1.79 (0.98–3.26)	0.057
Hb^a^ < 10 g/dL	1.71 (1.03–2.87)	0.040		
Type of VTE				
DVT	Reference	-		
PE	0.90 (0.51–1.58)	0.709		
Both DVT and PE	1.76 (0.90–3.44)	0.099		

^a^Missing data were handled via MICE; pooled estimates were calculated using Rubin’s rules. ^b^Prior clinically relevant bleeding within 3 months before anticoagulation initiation. ^c^Multivariable Fine–Gray subdistribution hazard model adjusted for age ≥ 65 years, BMI < 20 kg/m^2^, ECOG performance status ≥ 2, antiplatelet use, and non-DOAC therapy. BMI: body mass index; VTE: venous thromboembolism; DVT: deep vein thrombosis; PE: pulmonary embolism; Hb: hemoglobin; DOAC: direct oral anticoagulant; ECOG: Eastern Cooperative Oncology Group; SHR: sub-distribution hazard ratio; CI: confidence interval.

In the multivariable model, four independent predictors of CRB were identified. Concomitant antiplatelet therapy was associated with the largest effect size (adjusted sub-distribution hazard ratio (aSHR), 3.48; 95% CI, 1.76–6.90), followed by ECOG performance status ≥ 2 (aSHR, 3.05; 95% CI, 1.34–6.94). BMI < 20 kg/m^2^ was also independently associated with CRB (aSHR, 2.50; 95% CI, 1.36–4.61), as was age ≥ 65 years (aSHR, 1.97; 95% CI, 1.12–3.45).

Use of non-DOACs was associated with a numerically higher risk of bleeding (aSHR, 1.79; 95% CI, 0.98–3.26), although this did not reach statistical significance.

Multivariable analyses of predictors associated with CRB and MB are presented in Supplementary Material 4 (jocmr.elmerjournals.com).

### Mortality

During follow-up, 81 patients (20.5%) in the overall cohort died. Among those who experienced CRB, 18 patients (30.0%) died; however, only one death (5.6% of deaths in the bleeding group) was adjudicated as directly attributable to bleeding.

In multivariable time-dependent Cox proportional hazards analysis for all-cause mortality, both CRNMB and MB were independently associated with an increased risk of death. CRNMB was associated with an adjusted hazard ratio (aHR) of 3.72 (95% CI, 1.55–8.92), whereas MB was associated with a substantially higher risk (aHR, 5.62; 95% CI, 2.91–10.85) (Table 6).

**Table 6:** Univariable and Multivariable Time-Dependent Cox Regression Analysis of All-Cause Mortality

Variable	Univariable model		Multivariable model^c^	
	Hazard ratio (95% CI)	P value	Adjusted hazard ratio (95% CI)	P value
Bleeding status (time-dependent)				
No bleeding	1.00 (Reference)	-	1.00 (Reference)	-
After CRNMB	4.38 (1.86–10.30)	0.001	3.72 (1.55–8.92)	0.003
After major bleeding	5.87 (3.13–11.00)	< 0.001	5.62 (2.91–10.85)	< 0.001
Age ≥ 65 years	1.50 (0.97–2.33)	0.066	1.52 (0.96–2.43)	0.075
Male	0.92 (0.58–1.46)	0.723		
BMI^a^ < 20 kg/m^2^	1.58 (0.91–2.74)	0.107		
ECOG^a^ ≥ 2	1.51 (0.58–3.95)	0.402		
Diabetes mellitus	1.15 (0.69–1.91)	0.588		
Hypertension	1.16 (0.74–1.81)	0.515		
Coronary artery disease	1.80 (0.66–4.91)	0.254		
Cerebrovascular disease	1.69 (0.74–3.88)	0.216		
Chronic liver disease/cirrhosis	1.78 (0.56–5.65)	0.327		
Chronic kidney disease	1.74 (0.90–3.37)	0.102	1.71 (0.85–3.42)	0.131
Peripheral arterial disease	1.02 (0.14–7.31)	0.987		
Active cancer	3.06 (1.93–4.86)	< 0.001	2.93 (1.83–4.68)	< 0.001
Antiplatelet use	1.21 (0.58–2.52)	0.605		
History of previous bleeding^b^	1.03 (0.33–3.28)	0.954		
Non-DOAC therapy	3.10 (1.60–6.03)	0.001	2.76 (1.41–5.38)	0.003
Hb^a^ < 10 g/dL	1.87 (1.20–2.93)	0.006		
Type of VTE				
DVT	1.00 (Reference)	-	1.00 (Reference)	-
PE	2.48 (1.48–4.16)	0.001	2.16 (1.28–3.66)	0.004
Both DVT and PE	1.38 (0.65–2.96)	0.403	1.08 (0.49–2.37)	0.844

^a^Missing data were handled via MICE; pooled estimates were calculated using Rubin’s rules. ^b^Prior clinically relevant bleeding within 3 months before anticoagulation initiation. ^c^Multivariable time-dependent Cox proportional hazards model adjusted for time-dependent bleeding status (CRNMB and major bleeding), age ≥ 65 years, chronic kidney disease, active cancer, non-DOAC therapy, and type of VTE. CRNMB: clinically relevant non-major bleeding; BMI: body mass index; VTE: venous thromboembolism; DVT: deep vein thrombosis; PE: pulmonary embolism; Hb: hemoglobin; DOAC: direct oral anticoagulant; ECOG: Eastern Cooperative Oncology Group; CI: confidence interval.

### Recurrent thrombosis

During anticoagulant therapy, six patients (1.5%) developed recurrent venous thrombosis (DVT, n = 2; PE, n = 4). The median time to recurrence was 0.76 years (IQR, 0.02–0.96). The 1-year cumulative incidence, accounting for death and CRB as competing risks, was 1.98% (95% CI, 0.72–4.44). The incidence rate was 1.20 (95% CI, 0.54–2.68) per 100 patient-years over 498.89 patient-years of follow-up.

### Sensitivity analysis

After excluding 32 patients who received anticoagulants at less than therapeutic doses, 363 patients remained for the sensitivity analysis. The 1- and 2-year cumulative incidences of CRB were 16.01% (95% CI, 11.80–20.78) and 19.91% (95% CI, 14.77–25.62), respectively. For MB, the 1- and 2-year cumulative incidences of first MB were 8.38% (95% CI, 5.55–11.92) and 10.68% (95% CI, 7.07–15.13), respectively.

Comparisons of predictors of CRB across the multiple-imputation, complete-case, and sensitivity analyses are presented in Supplementary Material 5 (jocmr.elmerjournals.com).

## Discussion

This study provides real-world data on the incidence and predictors of anticoagulant-associated bleeding in a Thai cohort of patients with VTE. The incidence rate of CRB was 12.02 per 100 patient-years, with first MB and first CRNMB occurring at rates of 6.41 and 5.61 per 100 patient-years, respectively. Using a competing-risk framework with death treated as a competing event, the 1-year cumulative incidence of CRB was 14.89%, with MB accounting for 7.89% during the first year after initiation of anticoagulation. Concomitant antiplatelet therapy was the strongest independent predictor of CRB, followed by poor performance status (ECOG ≥ 2). In time-dependent multivariable Cox proportional hazards analysis, CRNMB was associated with an increased risk of all-cause mortality (aHR, 3.72), whereas MB conferred an even greater mortality risk (aHR, 5.62). These findings highlight the substantial clinical significance of anticoagulant-associated bleeding among Thai patients with VTE.

In the GARFIELD-VTE registry [6], a large international prospective cohort study, the incidence rate of any bleeding among Asian patients was 9.6 per 100 person-years (95% CI, 8.1–11.3), with MB occurring at 3.5 per 100 person-years (95% CI, 2.7–4.6). Although our study demonstrated numerically higher rates of CRB and MB, the overlapping CIs suggest no statistically significant difference. Notably, bleeding rates in VTE appear substantially higher than those reported in AF; a Thai cohort of patients with AF receiving oral anticoagulation reported an incidence rate of any bleeding of 2.37 per 100 patient-years (95% CI, 1.95–2.86) [9].

The relatively high bleeding rates observed in our study may partly reflect differences in patient characteristics, particularly the higher prevalence of active cancer compared with the GARFIELD-VTE Asian cohort (43.5% vs. 19.8%). However, active cancer was not independently associated with bleeding in our analysis, possibly because its effect was captured by other correlated factors, such as poor performance status and comorbidity burden. In addition, as a tertiary-care referral center, our institution is more likely to manage patients with complex clinical conditions who are at intrinsically higher risk of bleeding. Furthermore, the choice of anticoagulant may have been influenced by confounding by indication, as physicians may preferentially prescribe specific anticoagulants according to individual patient characteristics and perceived bleeding risk. Differences in the distribution of primary cancer sites across VTE cohorts may also have contributed to the observed variation in bleeding rates.

In our cohort, both CRB and MB occurred predominantly during the first year of anticoagulation. Beyond 2 years, the cumulative incidence of MB appeared to plateau, whereas CRB continued to increase gradually, suggesting that CRNMB may accumulate over time while MB occurs mainly during the early treatment phase. This pattern is consistent with prior studies demonstrating that bleeding risk is highest during the initial months of anticoagulant therapy [21].

As this was a retrospective study, interpretation of MB events may be challenging because event classification relies on documentation by treating physicians at the time of care. Therefore, we focused primarily on CRB, which combines MB and CRNMB and may provide a more robust outcome measure in retrospective analyses where misclassification between MB and CRNMB can occur.

Overall, the predictors of anticoagulant-associated bleeding identified in our cohort were broadly consistent with those reported in previous studies. In particular, advanced age has been consistently associated with a modest to moderate increase in bleeding risk across multiple studies of patients with VTE receiving anticoagulant therapy [21, 22].

Several factors previously associated with bleeding, including prior bleeding, cancer, CKD, and prior stroke [21–24], were not retained in the final multivariable model, possibly due to collinearity with other covariates. For example, most patients with coronary artery disease (10 of 11) and cerebrovascular disease (12 of 18) in our cohort were receiving concomitant antiplatelet therapy, which likely captured much of the associated bleeding risk. Similarly, CKD is strongly correlated with advanced age and poor performance status, which may have attenuated its independent effect in our analysis.

An important finding of our study was that BMI < 20 kg/m^2^ was an independent predictor of anticoagulant-associated bleeding. Although low BMI has been recognized as a bleeding risk factor in Asian populations, most evidence derives from studies in AF rather than VTE, and data in VTE remain limited [25–27]. Nevertheless, findings from a Japanese registry have demonstrated significantly higher 5-year cumulative incidences of MB and all-cause mortality among patients with lower body weight [27]. Several mechanisms may explain this association, including higher effective drug exposure, altered pharmacokinetics in individuals with low BMI, and the potential contribution of frailty or poor nutritional status [25, 28].

Given concerns about bleeding risk in Asian populations, reduced anticoagulant dosing is often used in clinical practice [23, 27, 29]. However, prior studies have not consistently demonstrated significant differences in bleeding risk across anticoagulant types or dosing strategies [30–32]. In our study, anticoagulant dosage was not included in the predictive analysis because the number of patients in each dosing category was too small for reliable evaluation.

GI bleeding accounted for a substantial proportion of bleeding events in our cohort. Consistently, concomitant antiplatelet therapy was identified as the strongest predictor of CRB. We did not collect data on proton pump inhibitor (PPI) prophylaxis in our study. PPIs are effective in reducing the risk of GI bleeding but are often underused in clinical practice [33, 34]. Future studies should evaluate the use of PPI prophylaxis in this population, including adherence to guideline recommendations and its potential role in reducing GI bleeding risk [35].

In time-dependent Cox regression analysis, non-DOAC use was associated with an increased risk of mortality. However, this finding should be interpreted with caution given the retrospective design of the study and the potential for confounding by indication. In Thailand, access to DOACs is largely restricted to certain reimbursement schemes, whereas many patients must receive warfarin. In addition, because CAT represented a large proportion of VTE patients in our cohort, LMWH was frequently used, which may also explain the relatively low use of DOACs.

Although only one death was directly attributable to bleeding, the magnitude of the mortality risk following bleeding events was substantial. This finding suggests that bleeding may trigger downstream clinical complications and increased hospitalization, ultimately contributing to worse overall outcomes.

The strengths of this study include a relatively large sample size for evaluating anticoagulant-associated bleeding specifically in patients with VTE. We applied competing-risk analysis to avoid overestimation of the cumulative incidence of CRB. In addition, time-dependent Cox regression was used to minimize immortal time bias. Missing data were handled using MICE, as the missingness pattern was consistent with a missing-at-random assumption, with acceptable imputation performance (fraction of missing information 0.38; relative efficiency > 99%).

This study has several limitations. First, it was conducted at a single tertiary-care referral center using a retrospective design, which may limit the generalizability of the findings and introduce selection bias. Second, missing data were unavoidable, and outcome misclassification may have occurred, particularly for MB events when complete information required to apply the ISTH criteria, such as the precise timing of hemoglobin decline, was unavailable. Third, although multivariable adjustment was performed, residual confounding cannot be excluded. In particular, confounding by indication may have influenced anticoagulant selection, as treatment decisions were made at the discretion of the treating physicians. Furthermore, data on warfarin time in therapeutic range (TTR) and anticoagulant drug levels were available only for a subset of patients and were therefore not included in the analysis.

To evaluate anticoagulant-specific predictors of bleeding, patients were censored at the time of switching to a different anticoagulant class, thereby reflecting the as-treated risk associated with each treatment. However, this approach may have introduced informative censoring. For example, if anticoagulant switching was prompted by early non-major bleeding or other clinical concerns, the subsequent risk of MB associated with the initial anticoagulant may have been underestimated.

Some baseline characteristics, such as smoking status, were inconsistently documented and were therefore missing for a substantial proportion of patients. In addition, several baseline coagulation parameters were not routinely measured before initiation of anticoagulant therapy at our institution. We acknowledge the absence of these baseline variables, which may have limited our ability to adjust for all potential confounders.

Finally, the study population was heterogeneous with respect to anticoagulant regimens, including variations in anticoagulant class and dosing, particularly among DOACs. Consequently, the sample size within individual dosing subgroups was insufficient to permit dose-specific regression analyses. This treatment heterogeneity may also have contributed to residual confounding.

Future studies should validate established bleeding risk prediction tools and compare their performance with newly developed models in prospective, multicenter cohorts. Particular attention should be given to the first 1–2 years after anticoagulant initiation, during which the risks of CRB and MB appear to be highest. In addition, the safety and effectiveness of different anticoagulant classes and dosing strategies should be evaluated prospectively, particularly in Southeast Asian populations, as bleeding risk profiles and treatment responses may differ from those reported in other Asian populations owing to differences in ethnicity, clinical characteristics, and healthcare practices.

In conclusion, anticoagulant-associated bleeding is common among Thai patients with VTE, particularly during the first year of treatment, and is independently associated with increased mortality. Concomitant antiplatelet therapy was the strongest predictor of bleeding, followed by poor ECOG performance status, low BMI (< 20 kg/m^2^), and advanced age. These findings highlight the importance of careful risk assessment and close monitoring during anticoagulant therapy in this population.

## Supplementary Material

Suppl 1 Flowchart of patient screening, eligibility assessment, and inclusion in the final analysis.

Suppl 2 Management of clinically relevant bleeding events.

Suppl 3 Anatomical sites of bleeding events categorized by antiplatelet use.

Suppl 4 Multivariable analysis of predictors associated with bleeding outcomes.

Suppl 5 Comparison of predictors of clinically relevant bleeding across multiple-imputation, complete-case, and sensitivity analyses.

## Data Availability

The datasets generated during and/or analyzed during the current study are available from the corresponding author on reasonable request.

## Abbreviations

AF: atrial fibrillation
aHR: adjusted hazard ratio
aSHR: adjusted subdistribution hazard ratio
BMI: body mass index
CAT: cancer-associated thrombosis
CI: confidence interval
CKD: chronic kidney disease
CNS: central nervous system
CRB: clinically relevant bleeding
CRNMB: clinically relevant non-major bleeding
CTPA: computed tomography pulmonary angiography
CTV: computed tomography venography
DOAC: direct oral anticoagulant
DVT: deep vein thrombosis
ECOG: Eastern Cooperative Oncology Group
EMR: electronic medical record
FMI: fraction of missing information
GI: gastrointestinal
ICD-10: International Classification of Diseases, Tenth Revision
ICH-GCP: International Conference on Harmonisation Good Clinical Practice
IQR: interquartile range
IRB: Institutional Review Board
ISTH: International Society on Thrombosis and Haemostasis
LMWH: low-molecular-weight heparin
MB: major bleeding
MCAR: missing completely at random
MICE: multiple imputation by chained equations
NSAID: nonsteroidal anti-inflammatory drug
OS: overall survival
PE: pulmonary embolism
PPI: proton pump inhibitor
REDCap: Research Electronic Data Capture
RVI: relative increase in variance
SD: standard deviation
TTR: time in therapeutic range
VKA: vitamin K antagonist
VTE: venous thromboembolism
