# Supplementary material for: Incidence and Predictors of Anticoagulant-Associated Bleeding in Patients With Venous Thromboembolism
Source: J Clin Med Res. 2026 Aug 26;18(8):585–98. doi: 10.14740/jocmr6679 (PMC13568748; doi:10.14740/jocmr6679)

Supplement Materials

S1. Flowchart of patient screening, eligibility assessment, and inclusion in the final analysis

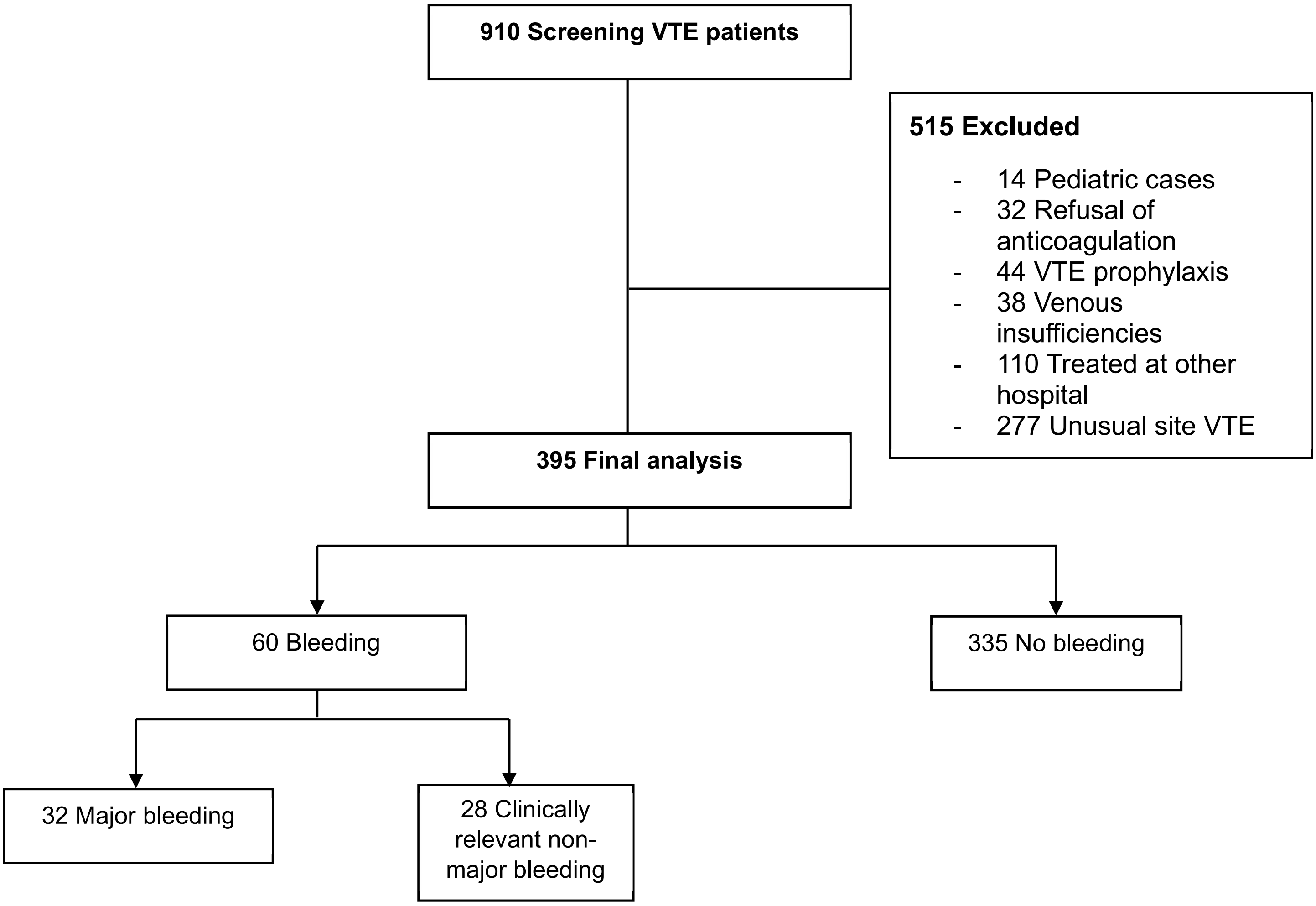

Supplement: Supplementary file 1 [file jocmr-18-08-585-s001.pdf]
