# Supplementary material for: Incidence and Predictors of Anticoagulant-Associated Bleeding in Patients With Venous Thromboembolism
Source: J Clin Med Res. 2026 Aug 26;18(8):585–98. doi: 10.14740/jocmr6679 (PMC13568748; doi:10.14740/jocmr6679)
Supplement: Supplementary file 2 [file jocmr-18-08-585-s002.pdf]

S2. Management of clinically relevant bleeding events

Management strategy	Total (N=60)	Major bleeding (N=32)	Clinically relevant non-major bleeding (N=28)	*P* value
**Packed red blood cell transfusion ≥2 units, n (%)**	28 (46.67)	28 (87.50)	0 (0.00)	<0.001
**Surgical hemostasis, n (%)**	24 (40.00)	19 (59.38)	5 (17.86)	0.001
**Inferior vena cava filter placement, n (%)**	4 (6.67)	3 (9.38)	1 (3.57)	0.616
**Definitive discontinuation of anticoagulation, n (%)**	31 (51.67)	20 (62.50)	11 (39.29)	0.120
**Reversal agent administered, n (%)**	15 (25.00)	14 (43.75)	1 (3.57)	<0.001
**Type of reversal agent***				
3-factor PCC	4 (26.67)	4 (28.57)	0 (0.00)	
Vitamin K	2 (13.33)	1 (7.14)	1 (100.00)	
Vitamin K and FFP	4 (26.67)	4 (28.57)	0 (0.00)	
Protamine	4 (26.67)	4 (28.57)	0 (0.00)	
aPCC	1 (6.67)	1 (7.14)	0 (0.00)	

* Percentages for reversal agent types were calculated using the number of patients who received reversal agents in each column as the denominator.

Abbreviations: FFP, fresh frozen plasma; PCC, prothrombin complex concentrate; aPCC, activated prothrombin complex concentrate
