# Supplementary material for: Incidence and Predictors of Anticoagulant-Associated Bleeding in Patients With Venous Thromboembolism
Source: J Clin Med Res. 2026 Aug 26;18(8):585–98. doi: 10.14740/jocmr6679 (PMC13568748; doi:10.14740/jocmr6679)
Supplement: Supplementary file 3 [file jocmr-18-08-585-s003.pdf]

S3. Anatomical sites of bleeding events categorized by antiplatelet use

Bleeding Site*	Total (n=60)	Concomitant Antiplatelet (n=12)	No Antiplatelet (n=48)	*P* value
Gastrointestinal (GI) tract				
Upper GI	16 (26.67%)	1 (8.33%)	15 (31.25%)	0.153
Lower GI	9 (15.00%)	4 (33.33%)	5 (10.42%)	0.069
Muscle / Soft tissue	16 (26.67%)	2 (16.67%)	14 (29.17%)	0.486
Central nervous system	6 (10.00%)	3 (25.00%)	3 (6.25%)	0.088
Genitourinary	7 (11.67%)	1 (8.33%)	6 (12.50%)	1.000
Respiratory tract	2 (3.33%)	0 (0.00%)	2 (4.17%)	1.000
Other sites	5 (8.33%)	1 (8.33%)	4 (8.33%)	1.000

* Patients could have bleeding at more than one site; percentages are based on the total number of patients in each group.
