# Supplementary material for: Incidence and Predictors of Anticoagulant-Associated Bleeding in Patients With Venous Thromboembolism
Source: J Clin Med Res. 2026 Aug 26;18(8):585–98. doi: 10.14740/jocmr6679 (PMC13568748; doi:10.14740/jocmr6679)
Supplement: Supplementary file 4 [file jocmr-18-08-585-s004.pdf]

S4. Multivariable analysis of predictors associated with bleeding outcomes

Predictor	All bleeding	*P* value	Major bleeding	*P* value
	**Adjusted SHR (95% CI)**		**Adjusted SHR (95% CI)**	
**Age ≥65 years**	1.97 (1.12-3.45)	0.018	1.44 (0.67–3.11)	0.354
**BMI <20 kg/m^2**	2.50 (1.36-4.61)	0.003	2.49 (1.11–5.63)	0.028
**ECOG ≥2**	3.05 (1.34-6.94)	0.008	2.45 (0.80–7.51)	0.115
**Antiplatelet use**	3.48 (1.76-6.90)	<0.001	1.82 (0.59–5.68)	0.300
**Non-DOAC use**	1.79 (0.98-3.26)	0.057	1.77 (0.73–4.32)	0.207

Abbreviations: BMI, body mass index; DOAC, direct oral anticoagulant; ECOG, Eastern Cooperative Oncology Group; SHR, sub-distribution hazard ratio; CI, confidence interval.
