# Supplementary material for: Incidence and Predictors of Anticoagulant-Associated Bleeding in Patients With Venous Thromboembolism
Source: J Clin Med Res. 2026 Aug 26;18(8):585–98. doi: 10.14740/jocmr6679 (PMC13568748; doi:10.14740/jocmr6679)
Supplement: Supplementary file 5 [file jocmr-18-08-585-s005.pdf]

S5. Comparison of predictors of clinically relevant bleeding across multiple-imputation, complete-case, and sensitivity analyses.

Variable	Multiple imputation (N=395)	*P* value	Complete-case analysis (N=242)	*P* value	Sensitivity analysis (N=363)	*P* value
	**aSHR (95% CI)**		**aSHR (95% CI)**		**aSHR (95% CI)**	
Age ≥65 years	1.97 (1.12-3.45)	0.018	2.32 (1.05–5.14)	0.039	2.07 (1.17–3.67)	0.013
BMI <20 kg/m^2	2.50 (1.36-4.61)	0.003	2.51 (1.11–5.69)	0.027	2.23 (1.19–4.16)	0.012
ECOG ≥2	3.05 (1.34-6.94)	0.008	4.98 (1.60–15.50)	0.006	3.17 (1.37–7.33)	0.007
Concomitant Antiplatelet	3.48 (1.76-6.90)	<0.001	1.86 (0.54–6.43)	0.327	2.86 (1.37–6.00)	0.005
Non-DOAC use	1.79 (0.98-3.26)	0.057	1.83 (0.79–4.27)	0.161	1.82 (0.90–3.65)	0.094

Abbreviations: BMI, body mass index; DOAC, direct oral anticoagulant; ECOG, Eastern Cooperative Oncology Group; aSHR, adjusted sub-distribution hazard ratio; CI, confidence interval
